# Allelic and Genotypic Distribution of *MMP13*-77 A/G Polymorphism in Salvadoran Children With and Without Caries

**DOI:** 10.3390/clinpract15080154

**Published:** 2025-08-19

**Authors:** Wendy Escobar-González, Jorge Alegría-Torres, Yolanda Terán-Figueroa, Vianney Castañeda-Monroy, Aurelio Álvarez-Vargas, Carlo Eduardo Medina-Solís, Nuria Patiño-Marín

**Affiliations:** 1 Doctoral Program in Dental Science, School of Dentistry, Autonomous University of San Luis Potosí, San Luis Potosí 78000, Mexico; wendy.escobar@ues.edu.sv (W.E.-G.); ja.alegriatorres@ugto.mx (J.A.-T.); yolandat@uaslp.mx (Y.T.-F.); cemedinas@uaeh.edu.mx (C.E.M.-S.); 2 Research Center, Faculty of Dentistry, University of El Salvador, San Salvador 1101, El Salvador; 3 Department of Pharmacy, University of Guanajuato, Guanajuato 36050, Mexico; 4 Health Research and Development Center, University of El Salvador, San Salvador 1101, El Salvador; vianney.monroy@ues.edu.sv; 5 Department of Biology, University of Guanajuato, Guanajuato 36050, Mexico; alvarez80@ugto.mx; 6 Academic Area of Dentistry, Health Sciences Institute, Autonomous University of Hidalgo State, Pachuca 42130, Mexico

**Keywords:** dental caries, genetic polymorphisms, metalloproteinases

## Abstract

**Background/Objectives**: Polymorphisms in metalloproteinases (MMPs) have the potential to be probable genetic biomarkers of dental caries. The aim of this study was to evaluate the distribution of the *MMP13* A/G rs2252070 single nucleotide polymorphism (SNP) according to caries experience in Salvadoran children. **Methods**: A cross-sectional study was carried out on 185 Salvadorian school children between 7 and 12 years of age. Demographic data, frequency of toothbrushing, dental flossing and consumption of sugar-sweetened beverages were recorded. Caries data were collected by clinical examination. Genomic DNA was obtained from oral cells of the children. Polymorphism genotyping was performed by PCR-RFLP. Allele and genotypic frequencies were compared between the healthy and caries-experiencing groups. Data were analyzed with SPSS 26.0 using the chi-square test, the Kruskal–Wallis test and logistic regression analysis. **Results**: The allele frequencies of *MMP13*-77 A/G were 0.7 and 0.3 following the Hardy–Weinberg equilibrium (X^2^ = 0.22, *p* = 0.63). 72% of subjects with caries experience were carriers of the A allele. Caries experience was higher for the GG genotype group for permanent and primary surfaces (DMFS = 2.11; dmfs = 5.64) and for permanent teeth (DMFT = 1.50). No significant differences were found in the allelic and/or genotypic frequencies of the SNP polymorphism between subjects with dental caries and healthy subjects (*p* > 0.05). **Conclusions**: The distribution of *MMP13*-77 A/G rs2252070 in the study population showed no association with caries experience. However, our findings highlight the importance of promoting oral hygiene habits from an early age.

## 1. Introduction

According to the World Health Organization, nowadays dental caries remains one of the most prevalent chronic diseases in the world [1]. The etiology of caries is multifactorial; it has been demonstrated that factors such as diet, inadequate exposure to fluoride, host habits and oral microbiota contribute to the appearance and severity of the carious process [2]. However, subjects exposed to very similar risk and protective factors may have substantial differences in susceptibility to caries [3]. In that sense, identifying genetic variations could contribute to a better understanding of individual susceptibility and, this way, support the development of strategies to prevent the disease in high-risk subjects [4].

It has been reported that genetic variations in enzymes known as metalloproteinases (MMPs) may be associated with vulnerability to caries [5,6,7]. The human MMP family includes 25 enzymes that have important roles in many biological and pathological processes. Collectively they are able to degrade practically all components of the extracellular matrix (ECM) [8]. Alterations in the expression of MMPs due to genetic polymorphisms could give rise to abnormal degradation of the ECM, leading to the development of different diseases [9,10]. Therefore, the study of SNPs in MMPs is receiving particular attention in the field of precision medicine. These are considered potential biomarkers for predicting the predisposition, status or progression of an individual’s disease [11].

At the level of dental structure, MMPs play an important role in the organization of the organic matrix of enamel and dentin [12]. These are found throughout the dentin, along the enamel–dentin junction and in the predentin [13,14]. In addition, there are other possible sources of MMPs in carious lesions: these are released in saliva by salivary glands, in crevicular fluid by cells in the gingival sulci, as well as in dentinal fluids by odontoblastic pulp cells [7,15,16].

Among the members of the multigene family of metalloproteinases, genetic variation in MMP13 has been among the most studied in relation to caries [17]. It is called collagenase-3 because it degrades components of the extracellular matrix such as collagens, gelatin, perlecan and fibronectin [18]. MMP13 is expressed during the budding stage of tooth development and is involved in bone development and repair [19]. It has been reported that the expression of MMP13 increases in dental tissue with the progression of caries [20,21]. It has been considered that variation in MMP13 expression could generate enamel that is more susceptible to high cariogenic challenges [22].

A functional polymorphism in the MMP13 gene is *MMP13*-77A/G (rs2252070), which has been associated with caries experience in other populations [23,24]. The aim of the present study was to evaluate the distribution of the *MMP13* rs2252070 single nucleotide polymorphism (SNP) according to caries experience and to explore the contribution of sociodemographic variables, dentobacterial plaque and oral hygiene habits in a population of children aged 7 to 12 years in El Salvador.

## 2. Materials and Methods

### 2.1. Study Design and Patients

A comparative cross-sectional study was conducted from February 2023 to November 2024 in El Salvador. This study was approved by the Ethics Committee of the Faculty of Dentistry of the University of El Salvador (approval number 2022-012), and the principles of the Declaration of Helsinki were complied with. Informed consent was obtained from parents or guardians as well as the assent of the study subjects. Healthy Salvadoran schoolchildren, 7–12 years of age and not related by blood, were studied. They had no relevant medical history and were not under antibiotic treatment.

### 2.2. Sample Size Calculation

The sample size was calculated using the Granmo program for comparing two independent proportions in a two-tailed test, assuming an alpha risk of 0.05 and a statistical power greater than 95%. An expected proportion of 0.335 was estimated for the caries-free group and 0.665 for the caries group, according to data reported by Çağırır et al. [23], who observed relevant differences in caries prevalence between genetic variants of MMPs. Under these assumptions, it was determined that 63 subjects per group were required to detect a statistically significant difference, also considering an expected substitution rate of 8%. The sampling technique was non-probability consecutive sampling.

### 2.3. Anamnesis

All parents or guardians answered a questionnaire about the study variables, including their children’s frequency of toothbrushing, flossing and consumption of sugary drinks.

### 2.4. Determination of Caries Experience

Intraoral examination was performed by the same dentist, using visual criteria and also a sterile dental probe and mirror to evaluate each subject. Dental plaque was assessed using a modified version of the Silness–Löe Plaque Index, which originally assigned scores from 0 to 3 based on the amount of plaque observed visually [25]. To facilitate epidemiological interpretation, these scores were dichotomized into ‘not visible’ (0–1) and ‘visible’ (2–3). Five surfaces of each tooth were examined and caries experience was assessed using the decayed, missing and filled index for both permanent and primary teeth (DMFT/dmft), as well as for surfaces (DMFS/dmfs), following the recommendations of the World Health Organization [26]. In addition, children were divided into two groups according to caries experience: healthy (subjects with dmft/DMFT = 0) or with caries (dmft/DMFT ≥ 1).

### 2.5. Allelic Discrimination

Genomic DNA for molecular analysis was extracted from buccal cells from the inner sides of the cheeks of each child, according to the modified method described by Mullot [27]. The quality of DNA samples was verified by UV spectrophotometry and gel electrophoresis. The SNP in MMP13 was analyzed by PCR-based restriction fragment length polymorphism analysis (PCR-RFLP). The primers used for amplification of the specific promoter region containing the *MMP13*-77A/G site were 5′-GATACGTTCTTACAGAAGAAGGC-3′ (forward) and 5′-GACAAATCATCTTCATCACC-3′ (reverse) [28].

Genomic DNA samples from each subject were mixed in a reaction with a final volume of 25 µL, containing 12.5 µL of GoTaq^®^ Green Master (Promega, Madison, WI, USA), 1 µL of 10 µM of each specific primer, 2 µL of each genomic DNA sample (25 ng/uL) and 8.5 µL of ultrapure endonuclease-free water. PCR conditions were an initial denaturation step at 94 °C for 5 min followed by 35 cycles at 94 °C for 1 min, 55 °C for 1 min, 72 °C for 1 min and a final extension step at 72 °C for 5 min. Subsequently, 5 µL of each PCR product was digested with the specific restriction enzyme Bsr I (New England Biolabs, Inc., Ipswich, MA, USA) for 2 h at 65 °C according to the manufacturer’s instructions. The products were analyzed on 2% agarose gels with 0.5 µL GelRed (Biotium, Fremont, CA, USA). The GG homozygote produced two bands of 248 bp and 197 bp, the AG heterozygote produced three bands, 445, 248 and 197 bp, and the AA homozygote produced a single band of 445 bp. Each sample was tested in duplicate.

### 2.6. Statistical Analysis

All statistical analyses were performed using the SPSS statistical package, version 26.0 (SPSS Inc., Chicago, IL, USA). The Hardy–Weinberg equilibrium X^2^ test was applied to determine the difference between observed and expected genotypic frequencies from allele frequencies. The Mann–Whitney test, X^2^ test and odds ratio calculation were used to analyze age, sex and preventive habits between the group with caries experience and the group without caries. The Kruskal–Wallis test was used to compare the means of caries indexes between genotype groups. A binary logistic regression analysis was adjusted for genotype, flossing and visible dentobacterial plaque to assess differences between the caries and caries-free groups. A *p*-value < 0.05 was considered statistically significant.

## 3. Results

A total of 185 individuals were included in this study; the mean age was 10.43 ± 1.43 years. A total of 53% were girls and 47% were boys. The mean DMFT and dmft were 1.19 ± 1.54 and 2.11 ± 2.56, respectively. A total of 61% of the schoolchildren had dental caries and 39% were healthy. Age and the presence of visible dentobacterial plaque were significantly associated with caries (Table 1).

The distribution of genotypic frequencies in the study population for *MMP13*-77 A/G was 50% homozygous AA, 40% heterozygous AG and 10% homozygous GG. The genotypes analyzed were in Hardy–Weinberg equilibrium: X^2^ = 0.22, *p* = 0.63. A higher frequency of subjects with caries experience were carriers of allele A compared to allele G; however the differences observed were not significant (Table 2).

On the other hand, higher caries indexes were observed in subjects with the GG genotype when we evaluated the surfaces and permanent teeth, as well as primary surfaces. After applying the Bonferroni correction to control for type I error in multiple comparisons between *MMP13* SNP genotypes, no statistically significant differences were found in caries experience indices. All adjusted *p*-values were equal to or greater than 0.972, and the 95% confidence intervals included zero, indicating that the differences observed between groups are not statistically significant (Table 3).

When the influence of the *MMP13*-77 A/G polymorphism and oral hygiene practices on dental caries experience was analyzed by binary logistic regression, only the presence of dentobacterial plaque showed a significant association (Table 4).

## 4. Discussion

Dental caries is a disease with a multifactorial nature and it is fairly well known that sociodemographic and environmental factors influence the development of caries [2]. However, it has been suggested that these factors are not sufficient to determine caries vulnerability in all individuals and that, in that sense, some genetic alterations could be potential biomarkers of predisposition to the disease [29]. This study was conducted to evaluate, for the first time in a Salvadoran population, the distribution of the *MMP13* rs2252070 polymorphism according to caries experience. Likewise, the influence of environmental factors in the healthy and carious groups was analyzed.

MMPs are known to have a fundamental role in enamel development, facilitating the orderly replacement of the organic matrix by minerals, which results in a structure with adequate properties [12,13]. In particular, MMP13 is expressed during early stages of tooth development, so it could be involved in the formation of enamel that is more resistant to demineralization [19]. On the other hand, during the caries process, the ECM of dentine—composed mainly of type I collagen—is exposed after mineral loss and becomes susceptible to hydrolytic degradation [7]. In this regard, MMP13, or collagenase-3, has the ability to degrade key components of the ECM [20,21]. Therefore, it has been suggested that alterations in MMP13 expression, such as those derived from genetic variations, could influence individual susceptibility to caries and its clinical progression [22].

*MMP13*-77 A/G rs2252070, located in the promoter region, appears to alter gene expression [24]. This SNP has been shown to be associated with some diseases such as osteoarthritis, vascular diseases and cancer [28,30,31]. At the level of the oral cavity, this polymorphism has been associated with periodontal disease, oral cancer, dental developmental defects and caries [22,32,33]. Therefore, the knowledge of the genetic distribution of rs2252070 in a population could make an important contribution to the identification of at-risk subjects and disease prevention.

This is the first report of the *MMP13*-77 A/G polymorphism in a population from El Salvador as well as the first attempt to analyze genetic factors related to caries experience in this country. According to our findings, the majority of subjects with caries experience belonged to the AA genotype; however, higher caries indexes were observed in subjects with the GG genotype for permanent teeth and surfaces as well as primary surfaces. The allele and genotypic frequencies for *MMP13*-77 A/G determined in this study are not similar to other reports on other populations [22,34,35,36]. However, allele frequencies reported in Turkey, Brazil and the Czech Republic are very similar to ours [23,24,37]. These studies included predominantly Caucasian or ethnically mixed Caucasian subjects.

There are conflicting results in the literature on the association between the *MMP13*-77 A/G polymorphism and caries experience. Çağırır et al. demonstrated that the *MMP13* A allele increases caries risk in a population of Turkish children and adolescents [23]. In turn, Tannure et al. reported that Brazilian carriers of the G allele have a significantly lower risk of caries [24]. According to the findings of the present study, *MMP13*-77 A/G is not related to caries in the Salvadoran population studied. These results are concordant with those reported by previous studies in other populations [22,34,35,36,37]. This variety of results among populations reveals the complex interaction between genetic variants and susceptibility to caries and reinforces the need to include underrepresented populations in genetic epidemiology to improve the generalization of findings and guide precision prevention strategies.

A systematic review and meta-analysis evaluated the association between the *MMP13* rs2252070 polymorphism and caries susceptibility across multiple populations. The authors concluded that this genetic variant may influence caries risk, although results varied depending on ethnicity and study design. These findings align with the biological plausibility of MMP13 involvement in enamel integrity and dentin degradation. However, in our Salvadoran cohort, no statistically significant association was observed, which may reflect population-specific genetic backgrounds or environmental modifiers. The inclusion of this evidence reinforces the need for regionally tailored studies and supports the relevance of exploring genetic markers in diverse populations [38].

This study classified caries experience according to the DMFT/dmft index, which facilitates epidemiological interpretation and comparisons between studies, but does not consider all stages of the disease. Future research could introduce analyses of caries severity thresholds or continuous outcomes to improve phenotyping resolution. It is essential to consider that the criteria for determining the diagnosis of dental caries may vary among studies that have used the DMFT/dmft. The incidence of caries may also vary due to each country’s health policies, demographics and ethnic factors [1]. In this sense, it is necessary to perform future studies with samples representative of the Salvadoran population’s demographics and to include other polymorphisms related to enamel formation, immune response and taste receptors.

As for the other factors, no differences in caries experience were found between boys and girls. However, a relationship with age was found, with a significantly lower risk in older schoolchildren. This could be due to the fact that these subjects predominantly had recently obtained permanent teeth in their oral cavity and were thus expected to be healthy. This result is consistent with those of other studies that included children and adolescents and applied a similar methodology to ours [23,24].

On the other hand, it is known that poor oral hygiene and frequent exposure to a cariogenic diet contribute to the development of caries due to intermittent fluctuations in salivary pH that lead to tooth demineralization [39,40]. According to our results, frequency of dental brushing, dental flossing and weekly consumption of sugar-sweetened beverages are not associated with caries risk. However, it was a limitation of the study that this information was obtained from the statements of parents or caregivers. For this reason, additionally, visible dental plaque was recorded as a clinical indicator of oral hygiene. In this regard, it was found that the presence of visible plaque on dental surfaces increased the risk of caries 2.88 times compared to those who did not present visible plaque during the oral examination.

In an acidic oral environment, which can occur due to poor oral hygiene and constant ingestion of cariogenic foods, host MMPs can be activated and are capable of destroying the dentin matrix. MMP13 has been identified to be expressed in dental pulp, healthy and decayed dentin and coronal and root caries [18,20]. It has been observed in extracted human teeth that its expression in dentin increases with caries progression [21]. In addition, it has been reported that MMP13 is expressed during the bud phase of tooth development in mice [41]. In view of this evidence, it follows that genetic variations in MMP13 could be linked to susceptibility to caries through different mechanisms, which could be assessed in other studies.

This study has limitations that should be considered when interpreting the findings. Although the sample size was adequate for general comparisons, it may have been insufficient to detect modest genetic effects, such as those previously reported [23,24]. In genetic association studies, power calculations must consider allele frequency and expected effect sizes to ensure statistical sensitivity. Additionally, it is important to emphasize that the sample came from a specific population, which may limit generalization. Replication in other populations is necessary to validate the observed associations and explore possible gene–environment interactions.

Although this study did not find a statistically significant association between *MMP13*-77 A/G and caries experience in the selected population, this result should be interpreted in the context of multiple factors that may influence the phenotypic expression of the disease. Dental caries is a multifactorial condition involving genetic, environmental, behavioral and microbiological variables, which may diminish the effect of individual variants. Furthermore, the absence of an association does not invalidate the relevance of the marker, but rather suggests the need for future studies with larger samples, greater statistical power, functional analyses and multigenic approaches. Reporting negative results helps to reduce publication bias and allows for the construction of a more robust and realistic base of evidence on the genetic determinants of caries.

## 5. Conclusions

Based on our results, we conclude that the distribution of the *MMP13*-77 A/G polymorphism showed no association with caries experience in the selected population. However, the tendency towards higher caries rates in GG genotype carriers could suggest a possible biological relevance. Likewise, the findings of this study highlight the importance of oral hygiene and the need to apply disease prevention strategies at an early age.

## Figures and Tables

**Table 1:** Sociodemographic variables, dentobacterial plaque and oral health habits of the study participants according to dental caries experience.

Variables	Caries Experience (*n* = 113)	Healthy (*n* = 72)	OR (CI 95%)	*p*-Value
Age, years, mean ± SD	10.26 ± 1.52	10.71 ± 1.23	-	0.03 ^1^
Sex, *n* (%)				
Male	53 (28.6)	34 (18.4)	1.0	0.96 ^2^
Female	60 (32.4)	38 (20.6)	1.01 (0.56–1.83)	
Tooth brushing frequency, *n* (%)				
3 times a day	27 (14.6)	17 (9.2)	1.0	0.97 ^2^
2 times a day	67 (36.2)	42 (22.7)	0.96 (0.66–1.40)	
1 time a day	19 (10.3)	13 (7)	1.00 (0.75–1.32)	
Flossing, *n* (%)				
Yes	6 (3.3)	7 (3.8)	1.0	0.25 ^2^
No	107 (57.8)	65 (35.1)	1.92 (0.61–5.96)	
Consumption of sugary drinks, *n* (%)				
1–3 times a week	70 (37.8)	47 (25.4)	1.0	0.64 ^2^
4 or more times a week	43 (23.3)	25 (13.5)	1.15 (0.62–2.13)	
Dentobacterial plaque, *n* (%)				
Not visible	93 (50.3)	67 (36.2)	1.0	0.03 ^2^
Visible	20 (10.8)	5 (2.7)	2.88 (1.02–8.06)	

*p* < 0.05, statistically significant; OR (95% CI) = Odds ratio, 95% confidence interval. ^1^ Mann–Whitney U test, ^2^ chi-square test.

**Table 2:** Allelic and genotypic distribution of *MMP13*-77 A/G polymorphism according to caries experience.

	Caries Experience (*n* = 113)	Healthy (*n* = 72)	Total (%)	*p*-Value
Allele, *n* (%)				
A	162 (72)	97 (67)	259 (70)	
G	64 (28)	47 (33)	111 (30)	0.37 ^1^
Genotype, *n* (%)				
AA	58 (51)	34 (47)	92 (50)	
AG	46 (41)	29 (40)	75 (40)	
GG	9 (8)	9 (13)	18 (10)	0.58 ^1^

*p* < 0.05, statistically significant. ^1^ Chi-square test.

**Table 3:** Genotypic distribution of *MMP13*-77 A/G polymorphism according to caries indexes.

Genotype	*n*	Surface Indexes				Teeth Indexes			
		dmfs	*p*-Value	DMFS	*p*-Value	dmft	*p*-Value	DMFT	*p*-Value
AA	92	4.38		1.77		2.13		1.26	
AG	75	4.31		1.41		2.10		0.99	
GG	18	5.64	0.507 ^1^	2.11	0.245 ^1^	1.79	0.761 ^1^	1.50	0.207 ^1^

Abbreviations: DMFS, Decayed, Missing and Filled Surfaces; DMFT, Decayed, Missing and Filled Teeth (dmfs/dmft for primary dentition; DMFT/DMFS for permanent dentition). *p* < 0.05, statistically significant. ^1^ Kruskal–Wallis Test. Note: Bonferroni correction, *p*-values adjusted ≥ 0.972.

**Table 4:** Binary logistic regression analysis of the association of the *MMP13*-77 A/G polymorphism and oral hygiene with dental caries.

Variable	OR (95% CI)	*p*-Value
Allele		
G	1.0	0.37
A	1.22 (0.77–1.92)	
Genotype		
GG	1.0	0.12 0.14
AG	2.4 (0.79–7.55)	
AA	2.3 (0.76–6.87)	
AA	1.0	0.59
GG + AG	1.17 (0.65–2.12)	
GG	1.0	0.31
AA + AG	1.65 (0.62–4.37)	
Flossing		
Yes	1	
No	1.55 (0.48–4.98)	0.46
Dentobacterial plaque		
No visible	1	
Visible	3.23 (1.08–9.60)	0.03

Significance level *p* ˂ 0.05.

## Data Availability

The data presented in this study are available on request from the corresponding author (N.P-M.) due to the fact that this information is protected by ethical regulations and by the Law for the Protection of Personal Data of El Salvador (in force since November 2024).

## Abbreviations

The following abbreviations are used in this manuscript:

MMP	Metalloproteinase
SNP	Single Nucleotide Polymorphism
DNA	Deoxyribonucleic Acid
PCR	Polymerase Chain Reaction
RFLP	Restriction Fragment Length Polymorphism
DMFT	Decayed, Missing and Filled Teeth
DMFS	Decayed, Missing and Filled Surfaces
ECM	Extracellular Matrix
SPSS	Statistical Package for the Social Sciences
OR	Odds ratio
CI	Confidence interval
